# Identification of COPZ1 as a Shared Candidate Ferroptosis‐Related Hub Gene in Periodontitis and Inflammatory Bowel Disease

**DOI:** 10.1155/humu/6316527

**Published:** 2026-09-12

**Authors:** Xin Yu, Yun Ruan, Zongying Zhang, Jiyuan Shi, Xinyu Gu, Yuyi Chen, Mengmeng Sang, Xiaorong Zhou, Yan Zhou, Liming Mao

**Affiliations:** ^1^ Department of Orthodontics and Periodontology, Affiliated Nantong Stomatological Hospital of Nantong University, Medical School of Nantong University, Nantong University, Nantong, China, ntu.edu.cn; ^2^ Department of Immunology, School of Medicine, Nantong University, Nantong, China, ntu.edu.cn; ^3^ Department of Gastroenterology, Affiliated Hospital of Nantong University, Nantong University, Nantong, China, ntu.edu.cn; ^4^ Jiangsu Key Laboratory of Oral Diseases, Nanjing Medical University, Nanjing, China, njmu.edu.cn; ^5^ Basic Medical Research Center, School of Medicine, Nantong University, Nantong, China, ntu.edu.cn; ^6^ Jiangsu Province Key Laboratory in University for Inflammation and Molecular Drug Target, Nantong University, Nantong, China, ntu.edu.cn

**Keywords:** COPZ1, Crohn′s disease, ferroptosis, periodontitis, ulcerative colitis

## Abstract

Clinical and epidemiological evidence demonstrates a robust bidirectional link between inflammatory bowel disease (IBD) and periodontitis, with IBD patients having a significantly elevated risk of periodontitis. Although this association is well established, the shared molecular mechanisms remain unclear. Both IBD, including Crohn′s disease (CD) and ulcerative colitis (UC), and periodontitis feature aberrant ferroptosis activation, which drives oxidative damage and tissue breakdown. We therefore hypothesized that ferroptosis is a core pathogenic mechanism common to all three conditions. Using GEO transcriptomic data, we identified 116, 134, and 255 exploratory candidate ferroptosis‐related differentially expressed genes in CD, UC, and periodontitis, respectively, with 55 candidate genes overlapping across all three conditions. Four machine learning–based feature selection algorithms consistently ranked COPZ1 as the top hub gene. In animal models of colitis and periodontitis, COPZ1 showed markedly elevated protein levels in inflamed intestinal and gingival tissues. In vitro experiments demonstrated that COPZ1 knockdown upregulated the expression of ferroptosis‐inhibitory genes (Gpx4, Slc7a11, and Fth1) in RAW264.7‐ASC macrophages under both basal and LPS‐induced inflammatory conditions, and reversed LPS‐mediated downregulation of Gpx4, suggesting that COPZ1 is involved in the regulation of ferroptosis in macrophages. Immune infiltration analysis revealed correlations between COPZ1 expression and the abundance of macrophages, neutrophils, B cell subsets, and T cell subsets, suggesting its association with differences in estimated immune cell composition. Single‐cell RNA sequencing from CD patient biopsies further demonstrated heterogeneous COPZ1 expression across diverse intestinal immune cell types (including neutrophils, CD8^+^ T cells, CD4^+^ T cells, and macrophages) and linked to ferroptosis‐associated intercellular signaling. In silico drug prediction identified nine small‐molecule compounds that stably bound to COPZ1 through hydrogen bonds. Molecular dynamics simulations subsequently ranked BRD‐K66453893 as the compound exhibiting the greatest structural stability among the tested candidates. Collectively, our findings identify COPZ1 as a shared candidate ferroptosis‐related hub gene for CD, UC, and periodontitis, and highlight its association with the inflammatory immune microenvironment, thereby providing a promising therapeutic target for these interrelated chronic inflammatory disorders.

## 1. Introduction

Inflammatory bowel disease (IBD) encompasses a group of chronic relapsing inflammatory disorders of the gastrointestinal tract, mainly including ulcerative colitis (UC) and Crohn′s disease (CD). These conditions are pathologically characterized by intestinal barrier damage, immune dysregulation, and persistent inflammation [1]. Periodontitis is a chronic infectious inflammatory disease affecting periodontal supporting tissues, including gingiva, periodontal ligament, and alveolar bone, clinically manifesting as gingival inflammation, progressive periodontal pocket formation, alveolar bone resorption, and, in advanced cases, tooth mobility or exfoliation. It represents one of the most prevalent chronic inflammatory diseases worldwide [2]. A growing body of clinical and epidemiological evidence supports a strong bidirectional comorbidity between the IBD and periodontitis. Patients with IBD exhibit a significantly elevated prevalence of periodontitis, with reported odds ratios (ORs) of up to 4.55 for UC and consistently elevated risk estimates for CD [3–5]. Conversely, periodontitis is associated with an increased incidence of IBD onset and heightened disease activity, potentially exacerbating intestinal inflammation through systemic immune activation [6, 7]. The oral–gut axis, mediated by translocation of oral pathogens to the gut, trafficking of activated immune cells, reciprocal modulation of microbial metabolites, and amplification of systemic inflammatory signaling, constitutes a key mechanistic framework underpinning this comorbidity [8, 9]. Nevertheless, despite the well‐established clinical and epidemiological overlap between IBD and periodontitis, the shared molecular pathways, particularly those governing epithelial integrity, innate immune sensing, inflammasome activation, and T cell polarization, remain incompletely defined and warrant systematic integrative investigation.

Ferroptosis is an iron‐dependent form of regulated cell death featured by lethal lipid peroxidation accumulation, which is widely implicated in various inflammatory diseases [10, 11]. In IBD, dysregulation of key ferroptosis‐related genes, including GPX4 [12], SLC7A11 [13], and ACSL4 [14], contributes critically to intestinal epithelial injury, mucosal barrier disruption, and subsequent amplification of inflammation. Pharmacological or genetic inhibition of ferroptosis effectively alleviates intestinal inflammation in preclinical models. Similarly, in periodontitis, aberrantly activated ferroptosis in periodontal ligament cells and osteoblasts exacerbates oxidative damage, impairs tissue homeostasis, and promotes bone resorption, while targeting ferroptotic molecules such as ALOX5 [15], NOX4 [16], and NRF2 [17] improves the periodontal inflammatory microenvironment and preserves periodontal architecture [18]. Given the shared hallmarks of chronic inflammation, progressive tissue destruction, and immune dysregulation in both conditions, it is plausible that IBD and periodontitis converge on common ferroptosis‐related hub genes and upstream regulatory networks.

The advancement of high‐throughput transcriptomic sequencing and advanced bioinformatics has enabled systematic interrogation of disease‐associated molecular signatures. In particular, machine learning–driven integrative analysis has emerged as a powerful approach for prioritizing biologically meaningful biomarkers and dissecting cross‐disease mechanisms from large‐scale transcriptomic data [19, 20]. Although increasing studies have independently investigated ferroptosis dysregulation in either periodontitis or IBD [21], a comprehensive, machine learning–informed identification of shared ferroptosis‐associated differentially expressed genes (DE‐FRGs) and their functional interactomes across these two disorders is still lacking, representing a critical knowledge gap that hinders the development of unified diagnostic criteria and mechanism‐based interventions for oral–gut comorbidity.

In the present study, we employed bioinformatics analysis and machine learning algorithms to identify shared DE‐FRGs consistently dysregulated in both periodontitis and colitis. We further delineated a core gene module, characterized its enrichment in biological pathways and immune cell infiltration patterns, and performed in silico drug molecule prediction coupled with molecular docking to prioritize clinically tractable therapeutic agents targeting the identified hub genes. Collectively, this study uncovers a systems‐level framework for understanding shared ferroptotic dysregulation in oral–gut inflammatory comorbidity and proposes novel, translationally relevant targets for synergistic diagnosis and targeted therapy.

## 2. Materials and Methods

### 2.1. Data Collection and Preprocessing

The gene expression data utilized in this study were all retrieved from the Gene Expression Omnibus (GEO) database, encompassing datasets related to CD, UC, and periodontitis. Specifically, the gene expression data for CD were derived from GSE20881 (99 cases vs. 73 controls) [22], GSE24287 (47 cases vs. 25 controls) [23], and GSE179285 (168 cases vs. 31 controls) [24]. For UC, the gene expression data were obtained from GSE13367 (34 cases vs. 20 controls) [25], GSE24287 (27 cases vs. 25 controls) [23] and GSE179285 (55 cases vs. 31 controls) [24]. Regarding periodontitis, the gene expression data were extracted from GSE23586 (3 cases vs. 3 controls) [26], GSE10334 (183 cases vs. 64 controls) [27] and GSE16134 (241 cases vs. 69 controls) [28]. In addition, 395 FRGs (Table S1) were obtained from the FerrDB repository (http://www.zhounan.org/ferrdb/). The CD, UC, and periodontitis datasets were separately merged, and the batch effects were corrected using the ComBat algorithm (v3.52.0) from the R package. PCA plots confirmed that the batch effects across different datasets were effectively rectified (Figure S1). Before correction, the PC1 values of periodontitis, UC, and CD datasets were 80.22%, 88.47%, and 71.53%, indicating severe batch effects; after ComBat correction, PC1 decreased to 15.87%, 12.77%, and 12.48%, with batch effects effectively removed and samples well mixed. The additional quantitative metrics of the pre‐ and postcorrection distribution results shown in the Table S2.

### 2.2. Identification of DE‐FRGs and Correlation Analysis Between Common Differentially Expressed Genes (Co‐DEGs)

Wilcoxon rank‐sum tests (*p* < 0.05, |log2FC| > 1) were applied to screen for candidate DE‐FRGs in CD, UC, and periodontitis tissue samples relative to controls. The resultant differential expression profiles were subsequently visualized as heatmaps via the “pheatmap” package. Pearson′s correlation analysis was performed to evaluate pairwise expression‐level correlations among Co‐DEGs and visualized by the “corrplot” package.

### 2.3. Screening of Core Diagnostic Biomarkers by Machine Learning

Four distinct machine learning approaches, including support vector machine (SVM), random forest (RF), generalized linear model (GLM), and eXtreme Gradient Boosting (XGB), were employed herein to evaluate the diagnostic value of Co‐DEGs for discriminating disease specimens from normal controls. Machine learning models were constructed using the caret package in R. A random seed was set to 123 to ensure reproducibility. The samples were randomly divided into a training set and an independent testing set at a ratio of 7:3 using createDataPartition, with stratification according to the outcome variable. Model training and internal optimization were performed on the training set using 5‐fold repeated cross‐validation via trainControl (method =  ^“^repeatedcv^”^, number = 5, and savePredictions = TRUE). Model performance was finally evaluated on the independent testing set. ROC curves and AUC values were generated from predicted probabilities for the held‐out testing samples rather than from the training set. For each algorithm, the Top 10 ranked genes were designated as candidate functional genes, and those identified as important by at least two of the four machine learning models were defined as core genes driving disease pathogenesis.

### 2.4. DSS‐Induced Colitis and Ligature‐Induced Periodontitis Mouse Models

Eight‐week‐old male C57BL/6 mice were used as experimental animals. All mice were housed under sterile isolated conditions with strictly controlled environmental parameters. Throughout the experiment, mice were given ad libitum access to standard chow. Mice were randomly divided into a control group and a DSS‐treated group [29]. Mice in the control group received distilled water ad libitum, whereas those in the DSS‐treated group were administered 3% dextran sulfate sodium dissolved in distilled water ad libitum for 7 consecutive days. For the periodontitis model [30], periodontitis was induced by ligating the right maxillary second molar with a 5‐0 silk suture, with the contralateral side left untreated as a control. After 7 days of ligation, mice were euthanized, and the maxillary bones were dissected and stripped of all soft tissues prior to microcomputed tomography (Micro‐CT) analysis. All experimental protocols and animal welfare practices were sanctioned by the Institutional Animal Care and Use Committee of Nantong University (Approval No. S20211204‐001).

### 2.5. Histological and Immunohistochemistry (IHC) Staining

After sacrifice, mice maxillary and colon tissues were dissected and immediately fixed in 4% paraformaldehyde. Maxillary tissues were further decalcified in 14% EDTA solution. All samples were subjected to gradient dehydration, transparency, and paraffin infiltration before embedding and sectioning. For hematoxylin–eosin (H&E) staining, tissue sections were stained using H&E stain kit (G1120, Solarbio, China). For IHC, heat‐mediated antigen retrieval was performed. Endogenous peroxidases were inactivated with H_2_O_2_ for 30 min, followed by blocking with PBS containing 5% BSA and 0.2% Triton X‐100 for 30 min at room temperature. Sections were incubated overnight at 4°C with primary antibody against COPZ1 (Affinity Biosciences, Cat. # DF3952). After rinsing with PBS, sections were incubated with appropriate secondary antibodies at room temperature. Immunoreactivity was visualized using DAB chromogen, and nuclei were counterstained with hematoxylin. All images were captured under a Leica DM4000 fluorescence microscope.

### 2.6. Cell Transfection, Inflammatory Modeling, and Real‐Time Fluorescent Quantitative PCR Detection

RAW264.7‐ASC cells were cultured routinely. When cells reached appropriate confluence, COPZ1‐specific small‐interfering RNA (siRNA) was transfected using CALNP RNAi in vitro transfection reagent (Cat. No. DN001‐05, D‐Nano, Beijing, China), with a nontargeting siRNA negative‐control group included. siRNAs targeting mouse COPZ1 were synthesized. Two independent siRNA duplexes were applied for knockdown efficiency validation: si‐COPZ1‐1 and si‐COPZ1‐2 (Table S3). For subsequent functional assays under LPS stimulation, only si‐COPZ1‐1 was used. A nontargeting negative‐control siRNA served as the negative control. After transfection, cells were treated with LPS for 12 h to establish an in vitro inflammatory cell model. Total RNA of each group was extracted by Trizol method, and qualified RNA samples were reversely transcribed into cDNA using HiScript III RT SuperMix for qPCR (Cat: R323‐01, Vazyme, Nanjing). Real‐time PCR was carried out with ChamQ Universal SYBR qPCR Master Mix (Cat: Q711‐02, Vazyme, Nanjing). *β*‐actin was selected as the internal reference gene. The primer sequences of COPZ1 and ferroptosis‐related genes were listed in Table S4. All experiments were conducted in triplicate. The relative mRNA expression levels of target genes were calculated by the 2^−△△CT^ method.

### 2.7. Immune Cell Infiltration Analysis

Immune‐cell infiltration across CD, UC, and periodontitis was systematically assessed using CIBERSORT in relative mode with the LM22 signature matrix and 1000 permutations. Quantile normalization was enabled (QN = TRUE), and deconvolution was performed on the ComBat‐adjusted merged expression matrices. The estimated fractions of the 22 immune‐cell populations were normalized to sum to one within each sample. Immune‐cell subsets exhibiting differential infiltration between disease and control groups were subsequently identified, and correlations between their estimated fractions and the expression levels of core ferroptosis‐related genes were further evaluated.

### 2.8. Analysis With Single‐Cell Sequencing Data

We analyzed the single‐cell RNA sequencing dataset GSE214695, which included 12 colonic samples with 6 control samples and 6 CD samples. A total of 46,700 cells passed the initial quality control and were processed using the Seurat R package (v5.3.0). All collected sample data were integrated into a single Seurat object. After integration, low‐quality cells were filtered out based on the percentage of mitochondrial RNA and the number of genes per cell. Subsequently, PCA was performed to assess the major sources of variation and determine the optimal number of principal components required for downstream analyses, followed by dimensionality reduction and visualization using Uniform Manifold Approximation and Projection (UMAP). In the single‐cell analysis, cells were classified according to whether COPZ1 transcripts were detected in the raw count matrix. Cells with COPZ1 count > 0 were defined as COPZ1‐detected cells, whereas cells with COPZ1 count = 0 were defined as COPZ1‐undetected cells. Because zero counts in scRNA‐seq data may be affected by technical dropout, this classification was interpreted as detection‐based rather than as definitive biological absence of COPZ1 expression. We firstly excluded cells with zero COPZ1 expression to minimize the confounding effects of technical dropout events. Then, we adopted the median expression level as a robust cutoff and reclassified cells into COPZ1 low‐expression and high‐expression groups. To validate the reliability and robustness of our COPZ1 grouping strategy, an independent external scRNA‐seq dataset (GSE134809) was downloaded from the GEO database. This dataset contains paired inflamed and uninflamed intestinal tissues derived from 11 individual patients with CD. Nevertheless, the paired‐sample design was not considered in our analytical workflow. The same quality control criteria, data integration, dimensionality reduction (PCA and UMAP), and median‐based COPZ1 grouping strategy were applied to this independent dataset. The FindMarkers function was used to identify differentially expressed genes. To explore intercellular communication, the CellChat package (v1.6.1) was applied to infer signal transduction interactions, which integrates ligand–receptor pairs with essential cofactors to construct a comprehensive intercellular communication network.

### 2.9. Drug Prediction, Molecular Docking, and Molecular Dynamics Simulations

The drug IC50 values were estimated using the oncoPredict package (v0.2) with data from the Cancer Therapeutics Response Portal (CTRP) based on the expression patterns of Co‐FRGs between normal and disease samples in CD, UC, and periodontitis. In addition, we analyzed the association between the predicted IC50 values of candidate drugs and Co‐DEG expression levels.

The receptor used for docking was the AlphaFold‐predicted structure of human COPZ1 isoform P61923‐2 (model AF‐P61923‐2‐F1), downloaded from the UniProt database. Because this predicted model contains no cocrystallized ligand, no ligand‐removal step was performed. Before docking, the downloaded model was prepared in UCSF Chimera (v1.18) by removing nonstandard bonds, and the processed structure was then used as the receptor for sphere generation and grid construction. Ligand structures were obtained from the ZINC15 and PubChem small‐molecule databases. Molecular docking was performed with DOCK6 using flexible‐ligand Anchor‐and‐Grow conformational sampling. The binding region was defined from receptor‐surface spheres generated with sphgen_cpp: sphere cluster 1 was selected, a docking box was generated around the selected spheres with showbox and van der Waals and electrostatic potential grids were calculated with grid. Docked poses were ranked by the DOCK6 Grid Score, defined as the sum of ligand–receptor van der Waals and electrostatic interaction terms. The reported values (−16.59 to −30.75) are energy‐based DOCK6 Grid Scores (nominally reported in kcal/mol) and should not be interpreted as experimental or calculated binding free energies. PyMOL v3.0.5 was used only for receptor‐ligand visualization.

Molecular dynamics simulations of the proteins and their complexes with ligands were performed using GROMACS package (v2023). Topologies for the proteins were generated with the AMBER99SB force field, whereas those for the ligands were prepared by the Chimera server. Structural properties, including the root mean square deviation (RMSD), radius of gyration (Rg), solvent‐accessible surface area (SASA), and root mean square fluctuation (RMSF) were analyzed. Trajectory visualization was conducted with CMD (v10.0.26100.2894), and the corresponding plots were generated using DuIvyTools software (v0.5.0).

### 2.10. Statistical Analysis

All statistical analyses were performed using R software (v4.4.2), with a *p* value < 0.05 defined as the threshold for statistical significance. For in vitro qPCR assays, two‐tailed Student′s *t*‐test was used for two‐group comparisons, whereas one‐way ANOVA with Bonferroni post hoc correction was adopted for multiple‐group comparisons. *n* refers to the number of biological replicates; technical replicates were conducted for each biological replicate to minimize intra‐assay experimental variation. Pearson′s correlation analysis was applied to evaluate gene–gene expression correlations among Co‐DEGs. Two‐sided Spearman′s rank‐correlation tests were used to assess associations between gene‐expression levels and immune‐cell fractions estimated by CIBERSORT. Groupwise differences in the estimated fractions of individual immune‐cell populations between disease and control cohorts were determined by two‐sided Wilcoxon rank‐sum tests. An empirical permutation test with 1000 permutations was implemented to evaluate the reliability of CIBERSORT deconvolution for each sample, and only samples with a CIBERSORT permutation *p* value < 0.05 were retained for subsequent downstream analyses.

## 3. Results

### 3.1. Identification of Common DE‐FRGs Across CD, UC, and Periodontitis

**Figure 1:**
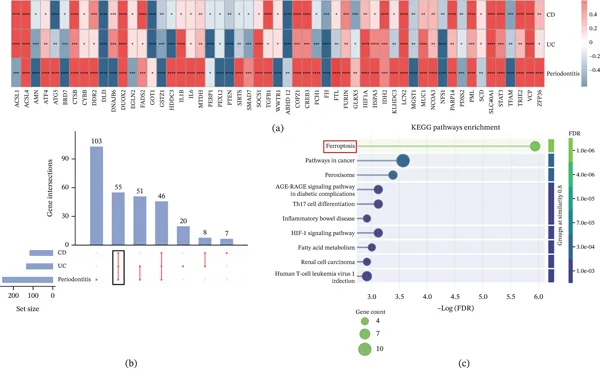
Identification of Co‐DEGs associated with CD, UC, and periodontitis. (a) Heatmap of the expression profiles of 55 Co‐DEGs in CD, UC, and periodontitis. (b) Upset plot illustrating the distribution of gene expression in multiomics data. (c) KEGG pathways enrichment plot of the 55 Co‐DEGs.

This study is aimed at systematically identifying DE‐FRGs shared across CD, UC, and periodontitis, thereby verifying whether dysregulated ferroptosis represents a common regulatory mechanism underlying the pathogenesis and progression of these three diseases. The gene expression profiles comprising colonic tissue samples from CD and UC patients, as well as gingival tissue samples from periodontitis patients, were retrieved from the GEO database. Each disease cohort was paired with anatomically and physiologically matched healthy control tissue samples (noninflamed colon for IBD and clinically healthy gingiva for periodontitis) to ensure biologically rigorous baseline comparisons in differential expression analysis. The analysis revealed substantial numbers of DE‐FRGs in each disease: 255 in periodontitis, 116 in CD, and 134 in UC. Further analysis revealed that 55 FRGs were differentially expressed across all three diseases (Figure 1a,b). KEGG pathway enrichment analysis of these 55 shared DE‐FRGs was subsequently conducted to explore their potential common regulatory pathways and biological processes implicated in their coordinated dysregulation (Figure 1c). Of note, the top‐ranked “Ferroptosis” KEGG term is a predictable outcome, as our input gene set was retrieved from the FerrDB database.

### 3.2. Correlation Analysis of the 55 Co‐DEGs

**Figure 2:**
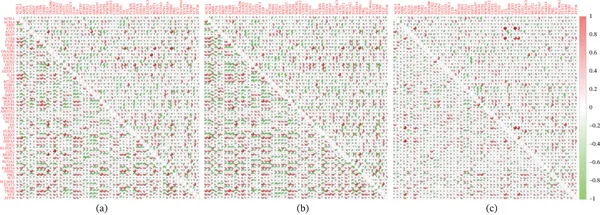
Correlation analysis of 55 Co‐DEGs in control group and disease group. (a) CD: merged GSE20881, GSE24287, and GSE179285. (b) UC: merged GSE13367, GSE24287, and GSE179285. (c) Periodontitis: merged GSE23586, GSE10334, and GSE16134.

To precisely analyze the possible interaction networks of the 55 Co‐DEGs in both healthy and diseased groups, we established the coexpression networks across independent cohorts of CD, UC, and periodontitis. For each cohort, heatmaps were stratified into two distinct quadrants: the disease subgroup (upper right) and the control subgroup (lower left). In CD (Figure 2a) and UC (Figure 2b), highly concordant expression patterns were observed: the control subgroups displayed robust positive and negative coexpression signals, marked by intense dark red and dark green signals, whereas the diseased subgroups showed markedly attenuated correlation magnitudes. This consistent attenuation suggests that the 55‐gene set is primarily preserved in homeostatic intestinal mucosa and is disrupted during active colitis, which may hint at a possible homeostasis‐maintaining rather than inflammation‐driving role in IBD pathophysiology. In contrast, the periodontitis cohort exhibited more prominent reversal pattern, with stronger and more extensive coexpression signals observed in the disease subgroup relative to controls (Figure 2c), possibly pointing toward the emergence of a tightly interconnected, dysregulated transcriptional network associated with periodontal inflammation. Collectively, these cohort‐stratified correlation analyses may reveal potential divergent functional rewiring of the same gene set across mucosal sites, potentially highlighting disease‐specific molecular signatures of FRGs in inflammatory pathogenesis, with correlational evidence for a pattern either as stabilizers of mucosal homeostasis in gut or as amplifiers of inflammatory dysregulation in oral tissue.

### 3.3. Machine Learning–Based Identification of Cross‐Disease Biomarker Candidates

**Figure 3:**
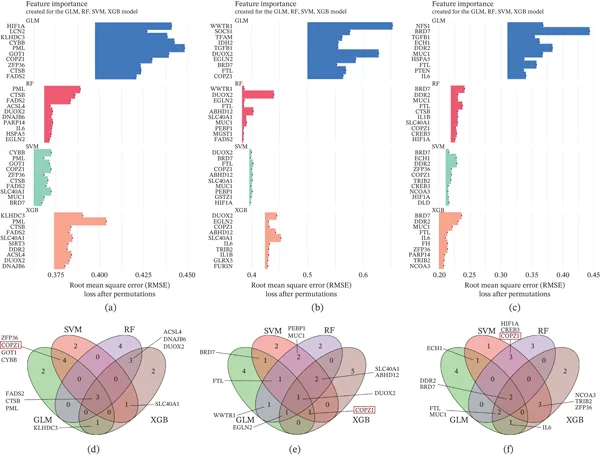
Important Co‐DEGs associated with CD, UC, and periodontitis as identified by machine learning algorithms. The Top 10 Co‐DEGs associated with (a) CD samples, (b) UC samples, and (c) periodontitis samples identified by GLM, RF, SVM, and XGB. Wayne diagram of important genes shared by two or more machine learning algorithms in (d) CD samples, (e) UC samples, and (f) periodontitis samples.

To identify robust, cross‐disease biomarker candidates among the 55 Co‐DE‐FRGs, we employed four complementary supervised machine learning models, GLM, RF, SVM, and XGB, to independently analyze feature importance in each disease cohort. Model performance was rigorously evaluated using ROC curve analysis, with all classifiers demonstrating strong discriminative capacity, thereby validating the reliability of gene prioritization (Figure S2). In the CD cohort (Figure 3a,d), COPZ1 emerged as a top‐ranked and consistently selected feature across all four models together with 11 additional genes forming a stable multimodel‐validated signature comprising 12 genes. In the UC cohort (Figure 3b,e), metabolism‐related genes dominated the top‐ranked features. Notably, COPZ1 and DUOX2 were jointly identified as high‐confidence shared biomarkers between CD and UC, indicating that the vesicular transport and oxidative stress pathways synergistically participate in the common pathological processes of IBD. In the periodontitis cohort (Figure 3c,f), BRD7 was identified as the most discriminative feature across all models. Importantly, COPZ1, MUC1, and FTL exhibited overlap in feature importance rankings between the periodontitis and UC model, thereby providing core molecular evidence for a mechanistic link underlying the clinical comorbidity between periodontitis and UC.

In addition, systematic intersection analysis of top‐performing features across cohorts further revealed COPZ1 as the sole characteristic gene exhibiting overlap across all three disease groups (CD, UC, and periodontitis). Specifically, COPZ1 and DUOX2 constituted the only intersecting features between CD and UC, whereas COPZ1 served as the central hub linking periodontitis and both IBD subtypes. These findings collectively position COPZ1 as a potential pan‐disease biomarker candidate for IBD‐periodontitis comorbidity, offering a theoretical foundation for developing integrated early‐screening strategies and rational multitarget therapeutic intervention.

### 3.4. The Expression of COPZ1 in Animal Models of Colitis and Periodontitis

**Figure 4:**
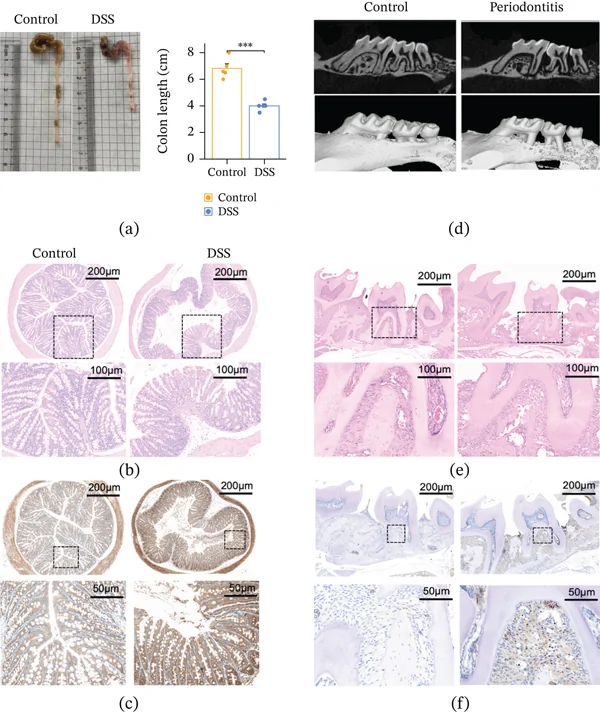
Establishment of mouse models of DSS‐induced colitis and ligature‐induced periodontitis, and expression of COPZ1 in these models. (a) Representative images of colon length in control and DSS‐treated mice. (b) H&E staining of colon tissues from control and colitis mice. (c) IHC staining of COPZ1 in colon tissues. (d) Micro‐CT analysis of alveolar bone in control and periodontitis mice. (e) H&E staining of periodontal tissues. (f) IHC staining of COPZ1 in periodontal tissues.

In the DSS‐induced colitis model, mice exhibited significantly shortened colon length relative to control animals (Figure 4a), consistent with severe intestinal inflammation and tissue injury. H&E staining revealed disrupted colonic mucosal architecture, including loss of crypt integrity, epithelial erosion, and massive inflammatory cell infiltration into the lamina propria, in colitis mice. In contrast, control mice displayed intact colonic mucosa with regular epithelial arrangement and minimal immune cell presence (Figure 4b). IHC staining demonstrated markedly elevated protein expression of COPZ1 in colonic tissues from colitic mice (Figure 4c).

In the ligature‐induced periodontitis model, Micro‐CT analysis showed prominent low‐density radiolucent lesions around the molar roots and furcation regions, accompanied by reduced alveolar ridge height and obvious alveolar bone resorption in periodontitis mice compared with sham‐operated controls (Figure 4d). H&E staining corroborated these structural changes, revealing obvious alveolar bone resorption and intensive perivascular and periosteal inflammatory cell infiltration in periodontitis mice (Figure 4e). Consistent with the colitis findings, IHC staining revealed markedly elevated COPZ1 expression in periodontal tissues of periodontitis mice, particularly in inflammatory cells of the gingival connective tissue and cells surrounding the alveolar bone (Figure 4f). Collectively, these findings demonstrate consistent upregulation of COPZ1 protein in inflamed intestinal and periodontal tissues across two distinct models of chronic inflammatory disease, suggesting a potential role for COPZ1 in mediating shared inflammatory mechanisms in the pathogenesis of both intestinal and periodontal inflammatory diseases.

### 3.5. COPZ1 Mediates Ferroptosis Regulation in Inflamed RAW264.7‐ASC Macrophages

**Figure 5:**
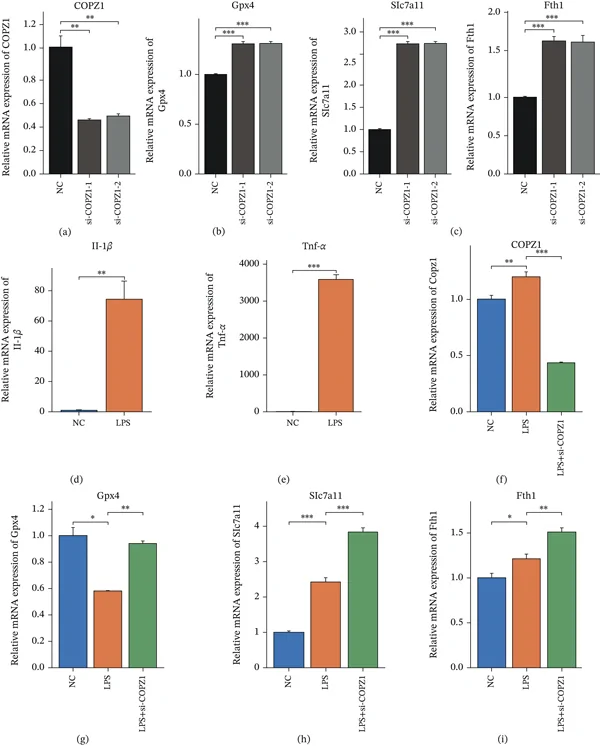
COPZ1 knockdown regulates ferroptosis‐related gene expression in RAW264.7‐ASC macrophages under basal and LPS‐induced inflammatory conditions. (a–d) qRT‐PCR validation of COPZ1 knockdown efficiency and changes in Gpx4, Slc7a11, and Fth1 expression. (e,f) LPS‐induced inflammation was confirmed by increased Il‐1*β* and Tnf‐*α* expression. (g–j) LPS‐stimulated cells were transfected with si‐COPZ1, and mRNA levels of COPZ1, Gpx4, Slc7a11, and Fth1 were detected by qRT‐PCR.  ^∗^ *p* < 0.05,  ^∗∗^ *p* < 0.01,  ^∗∗∗^ *p* < 0.001. *n* = 3 biological replicates.

To explore the regulatory effect of COPZ1 on macrophage ferroptosis, we silenced COPZ1 in RAW264.7‐ASC cells using siRNA. Two distinct interfering sequences effectively decreased COPZ1 transcription (Figure 5a). After COPZ1 knockdown, the expression of anti‐ferroptotic genes Gpx4, Slc7a11, and Fth1 was markedly increased (Figure 5b–d), indicating suppressed ferroptotic progress.

An LPS‐induced inflammatory model was established. Elevated proinflammatory Il‐1*β* and Tnf‐*α* confirmed valid inflammatory stimulation (Figure 5e,f). LPS elevated COPZ1 expression and restrained Gpx4 transcription. In addition, COPZ1 depletion reversed such alterations, restored Gpx4 expression and further raised levels of Slc7a11 and Fth1 (Figure 5g–j). These results indicated that COPZ1 participates in ferroptosis regulation in macrophages under normal and inflammatory states.

### 3.6. Evaluation of COPZ1′s Association With Immune Cell Infiltration Across Inflammatory Diseases

We systematically evaluated the association between COPZ1 expression and immune cell infiltration patterns across three inflammatory diseases using butterfly charts. In healthy control cohorts across all three diseases, COPZ1 exhibited weak and statistically nonsignificant correlations with the majority of immune cell subsets, suggesting that its regulatory role in immune cell infiltration is negligible under physiological conditions.

**Figure 6:**
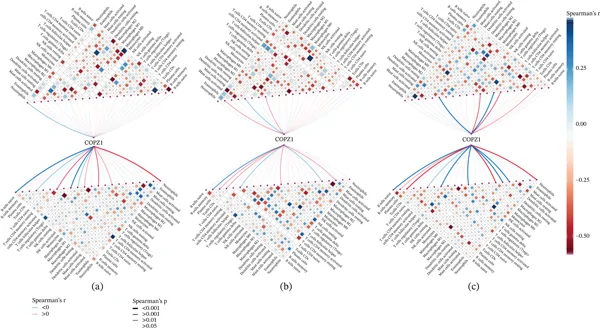
The correlation analysis between COPZ1 expression and immune cell infiltration patterns in CD, UC, and periodontitis. Butterfly plots showing the Spearman′s correlation between COPZ1 expression and immune cell infiltration levels in control (upper panels) and disease (lower panels) groups across three cohorts: (a) CD, (b) UC, and (c) periodontitis.

In contrast, the strength and statistical significance of the correlations between COPZ1 expression and immune cell infiltration increased markedly in disease groups. In CD patients (Figure 6a), COPZ1 showed significant positive and negative correlations with multiple immune cells, particularly B cells, T cells, and neutrophils, indicating its involvement in shaping intestinal inflammatory immune imbalance. In UC patients (Figure 6b), COPZ1 exhibited a selective and statistically robust association, predominantly with memory B cells, while showing no significant correlations with T cell subsets or neutrophils. This distinct immunological signature, divergent from the broader immune cell associations observed in CD, underscores a disease‐specific regulatory role for COPZ1 in UC pathogenesis. In periodontitis patients (Figure 6c), correlations between COPZ1 and immune cells, especially neutrophils, macrophages, B cell subsets, and T cell subsets were more prominent, implying that COPZ1 may participate in periodontal inflammation by regulating the infiltration of specific immune cells. Cross‐disease comparisons revealed that enhanced associations between COPZ1 and immune infiltration represent a common feature across these inflammatory diseases, whereas the identity, directionality, and effect size of individual correlations exhibit disease‐specific patterns. This duality suggests that COPZ1 functions not as a generic immune activator, but rather as a context‐dependent modulator whose regulatory impact is shaped by the local inflammatory microenvironment.

### 3.7. The Role of COPZ1 in the Regulation of the Immune Microenvironment and Intercellular Communication Through scRNA‐Seq Analysis

**Figure 7:**
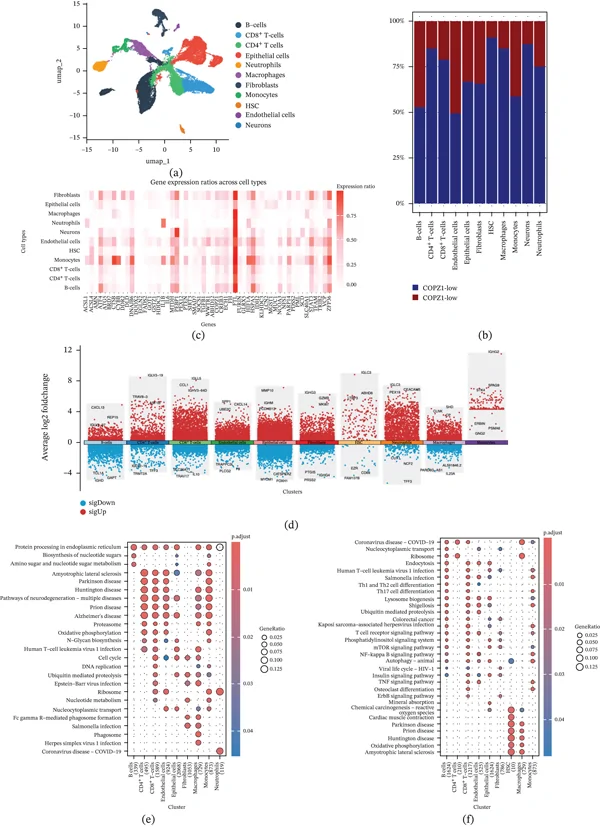
ScRNA‐seq analysis. (a) Cell type annotation. (b) Bar plot showing the distribution of COPZ1‐high and COPZ1‐low cells across clusters. (c) Heatmap illustrating the distribution of 55 Co‐DEGs across different cell types. (d) Scatter plot of differential expression analysis between COPZ1‐high and COPZ1‐low cells. (e) KEGG pathway enrichment of upregulated DEGs. (f) KEGG pathway enrichment of downregulated DEGs.

Herein, we leveraged the publicly available scRNA‐seq dataset GSE214695, comprising 12 colonic tissue samples from 6 healthy donors and 6 patients with CD, to systematically elucidate the regulatory role of COPZ1 in the intestinal immune microenvironment. Based on UMAP dimensionality reduction and clustering analysis, 11 transcriptional distinct cell types, including B cells, T cells, fibroblasts, epithelial cells, monocytes, and macrophages, were identified from the samples (Figure 7a). Across all analyzed cell types, COPZ1‐low expressing cells dominated the composition, whereas COPZ1‐high expressing cells represented a minor proportion in most populations. Notably, B cells and endothelial cells harbored relatively higher fractions of COPZ1‐high cells, whereas the proportion of COPZ1‐high cells was low in neutrophils, CD8^+^ T cells, CD4^+^ T cells, and macrophages (Figure 7b), illustrating heterogeneous COPZ1 expression across diverse cell populations. To further validate the reliability of our median‐based COPZ1 grouping strategy, we additionally performed external validation using an independent scRNA‐seq dataset GSE134809, with highly consistent results observed (Figure S3). The gene expression heatmap further uncovered heterogeneous expression patterns of 55 co‐DE‐FRGs across all annotated cell types. Notably, FTL was highly expressed in all 11 cell types (Figure 7c). Differential gene expression analysis between COPZ1‐high and COPZ1‐low cells within each cluster demonstrated that COPZ1 expression status could regulate the transcriptional profiles of each cell cluster (Figure 7d). For instance, in B cells, genes such as CXCL13 were upregulated, whereas genes including IGHD were markedly downregulated. Functional pathway enrichment analysis indicated that COPZ1‐associated DEGs were mainly enriched in immune, metabolic, and protein processing‐related pathways such as the T cell receptor signaling pathway, NF‐*κ*B signaling pathway, oxidative phosphorylation, ribosome function, and endoplasmic reticulum protein processing (Figure 7e,f). These findings suggest that COPZ1 may be involved in the pathological processes of immune disorder and metabolic abnormality in CD by regulating immune cell infiltration and the activation of functional pathways.

**Figure 8:**
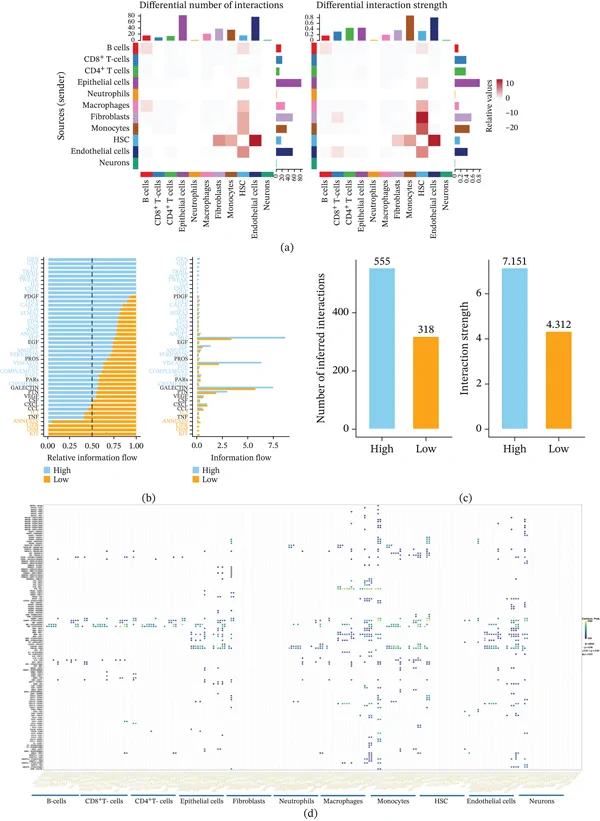
Cell–cell communication between COPZ1‐high and COPZ1‐low cells. (a) Differential interactions between cell types. (b) Bar plot comparing the inferred number and strength of interactions between COPZ1‐high and COPZ1‐low cells. (c) Visualization of differences in molecular information flow. (d) Bubble plot of cell–cell communication.

To further dissect its role in intercellular coordination, we performed ligand–receptor‐based cell–cell communication inference using CellChat. Compared with the COPZ1‐low group, the COPZ1‐high group exhibited markedly enhanced communication activity, particularly involving fibroblasts, monocytes, and endothelial cells, which manifested as increased numbers of ligand–receptor interactions and higher interaction scores (Figure 8a). Pathway‐specific information flow analysis further demonstrated that the COPZ1‐high group was dominated by pathways such as GRN, PDGF, and IL‐1 in terms of relative information flow, whereas the COPZ1‐low group was centered on OSM, LIFR, and annexin signaling pathways. Absolute information flow analysis confirmed that factors including MIF, VISFATIN, and GALECTIN displayed higher signal transmission efficiency in the COPZ1‐high group, indicating that COPZ1 can positively regulate pathways associated with immune responses and stromal cell activation (Figure 8b). Quantitative validation results showed that the COPZ1‐high group had higher numbers of inferred interactions (555 vs. 318) and greater average interaction strength (7.151 vs. 4.312) (Figure 8c). Finally, we compared the cell–cell communication patterns of COPZ1‐high and COPZ1‐low cells using a bubble plot representation of intercellular interactions (Figure 8d).

### 3.8. Drug Prediction, Molecular Docking, and Molecular Dynamics Simulation Targeting COPZ1

**Figure 9:**
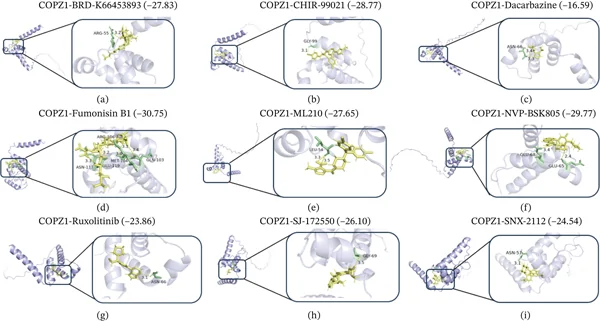
Molecular docking analysis. (a) COPZ1 bound with BRD‐K66453893. (b) COPZ1 bound with CHIR‐99021. (c) COPZ1 bound with dacarbazine. (d) COPZ1 bound with fumonisin B1. (e) COPZ1 bound with ML210. (f) COPZ1 bound with NVP‐BSK805. (g) COPZ1 bound with ruxolitinib. (h) COPZ1 bound with SJ‐172550. (i) COPZ1 bound with SNX‐2112.

To identify candidate therapeutics with potential pan‐disease activity in CD, UC, and periodontitis, we first performed differential expression analysis of the 55 FRGs across disease‐relevant tissue transcriptomes (Figure S4). This prioritized 17 candidate drugs exhibiting common regulatory effects on these three diseases. Further molecular docking analysis between COPZ1 (the top‐ranked, cross‐disease–associated FRG) and these 17 candidates yielded nine compounds with favorable DOCK6 Grid Scores and predicted hydrogen‐bond contacts with COPZ1 (Figure 9a–i). Among these, fumonisin B1 had the most favorable (most negative) Grid Score (−30.75), whereas dacarbazine had the least favorable score (−16.59). These values are DOCK6 energy‐based Grid Scores rather than binding free energies. Pose inspection predicted that Fumonisin B1 formed five hydrogen‐bond contacts with ARG106, GLU119, MET104, GLN103, and ASN117. These docking poses provide testable structural hypotheses for subsequent experimental validation rather than direct evidence of binding affinity.

**Figure 10:**
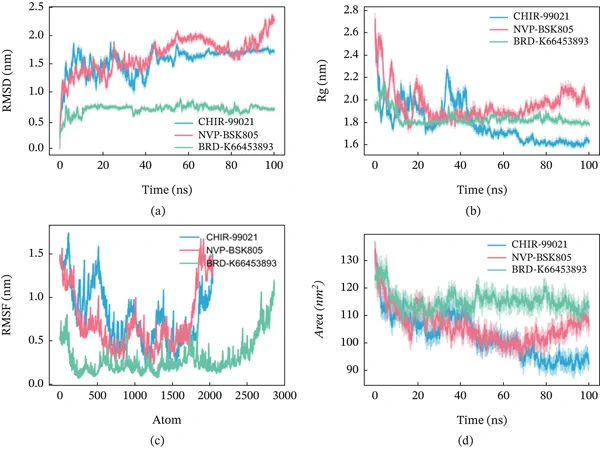
Molecular dynamics simulations of COPZ1 in complex with CHIR‐99021, NVP‐BSK805, and BRD‐K66453893. Panels show (a) RMSD, (b) Rg, (c) RMSF, and (d) SASA analysis.

In addition, we compared the interactions of COPZ1 with the Top 4 drugs exhibiting the lowest docking scores: fumonisin B1, BRD‐K66453893, NVP‐BSK805, and CHIR‐99021. Fumonisin B1 was excluded due to possible uncertainties in its chemical structure. Molecular dynamics simulations revealed differences in conformational stability among COPZ1 complexes with three distinct small‐molecule compounds. RMSD analysis demonstrated that the BRD‐K66453893 group exhibited the smallest structural deviation and highest temporal stability, whereas the NVP‐BSK805 group displayed the largest fluctuation (Figure 10a). Rg analysis indicated that the COPZ1 structure was most compact in the BRD‐K66453893 system, whereas it became relatively loose in the NVP‐BSK805 group (Figure 10b). RMSF results suggested that local flexibility of COPZ1 was markedly suppressed by BRD‐K66453893 but enhanced in the NVP‐BSK805 group (Figure 10c). Consistent with these findings, SASA analysis further verified the optimal structural compactness and stability in the BRD‐K66453893 complex (Figure 10d). Collectively, multiparameter MD validation identifies BRD‐K66453893 as the structurally stable small‐molecule modulator of COPZ1 among the three tested compounds.

## 4. Discussion

In the present study, we identified 116, 134, and 255 FR‐DEGs in CD, UC, and periodontitis, respectively. Integrated cross‐disease analysis revealed a core set of 55 overlapping FR‐DEGs, among which COPZ1 emerged as the top‐ranked consensus hub gene, consistently prioritized across four complementary machine learning algorithms. Validation in animal models confirmed upregulation of COPZ1 protein expression in inflamed colonic tissues (DSS‐induced colitis) and periodontal tissues (ligature‐induced periodontitis), supporting its association with chronic inflammation along the gut–oral axis. Given the established role of dysregulated ferroptosis in driving epithelial barrier disruption, immune cell death, and tissue injury in both IBD and periodontitis [31, 32], shared molecular regulators such as COPZ1 may provide novel insights and promising candidates for deciphering mechanistic links underlying their clinical comorbidity. To our knowledge, this is the first study to identify COPZ1 as a cross‐tissue, cross‐disease hub gene associated with intestinal and periodontal inflammation, thereby providing a novel, targetable node for understanding shared pathogenic mechanisms in chronic inflammatory diseases.

COPZ1 encodes the zeta 1 subunit of the coatomer protein complex I (COPI), a canonical mediator of vesicle transport between the endoplasmic reticulum and Golgi apparatus [33]. Emerging evidence has expanded its functional repertoire to include critical roles in regulating intracellular iron homeostasis and ferroptosis. In vitro experiments revealed that COPZ1 knockdown altered the expression of key ferroptosis‐related genes, including Gpx4, Slc7a11, and Fth1, in RAW264.7‐ASC macrophages under both basal and LPS‐induced inflammatory conditions, and restored LPS‐mediated Gpx4 downregulation, suggesting that COPZ1 is involved in the regulation of ferroptosis in macrophages. Previous studies have reported cell‐ and context‐dependent roles of COPZ1 in ferroptosis regulation. In lung adenocarcinoma, COPZ1 mediates ferritinophagy by regulating transcription and posttranslational stabilization of NCOA4, thereby suppressing ferroptosis sensitivity, and promoting tumor proliferation and chemoresistance [34]. In glioblastoma, elevated COPZ1 expression is positively correlated with advanced tumor malignancy and poor prognosis. Conversely, silencing COPZ1 induces ferroptosis, impairs tumor growth, and enhances anti‐tumor immune responses [35]. Collectively, COPZ1 presents diverse functions in oncologic and inflammatory disorders. Our results showing high COPZ1 expression in CD, UC, and periodontitis, three chronic mucosal inflammatory disorders, further support its participation in common pathogenic processes, making it a promising therapeutic target for multiple diseases.

Dysfunction of the immune microenvironment is a core pathological feature of chronic inflammatory diseases, driven primarily by imbalanced immune cell infiltration and disordered intercellular communication, both of which perpetuate inflammation and exacerbate tissue damage. Our bulk transcriptomic immune infiltration analysis demonstrated statistically significant correlations between COPZ1 expression and the infiltration levels of key innate and adaptive immune populations, including macrophages, neutrophils, B cell subsets, and T cell subsets, suggesting an association of COPZ1 with the cellular composition and functional status of the inflammatory microenvironment. Consistent with this, single‐cell RNA sequencing analysis in CD revealed heterogeneous COPZ1 expression across intestinal immune cell populations, including neutrophils, CD8^+^ T cells, CD4^+^ T cells, and macrophages. Moreover, ligand–receptor interaction modeling demonstrated that COPZ1 status participates in cell–cell communication and signal transduction networks, showing potential links to the activation, recruitment dynamics, and context‐dependent signal transmission of immune cells in the inflammatory microenvironment. Integrating these findings with established roles for COPZ1 in ferroptosis regulation, including its control of ferritinophagy and iron metabolism, we propose an underlying mechanistic framework: COPZ1 functions at the nexus of ferroptotic stress and immune dysregulation, where its upregulation concurrently impairs redox homeostasis and reprograms immune cell behavior. This ferroptosis–immune axis may therefore represent a convergent pathogenic driver across chronic inflammatory diseases.

In addition, we performed virtual screening targeting the COPZ1 protein structure and obtained nine candidate compounds that form stable, high‐probability hydrogen bond interactions with key residues in the predicted binding pocket. Subsequent molecular dynamics simulation verified that BRD‐K66453893 exhibited the most stable conformation, indicating its potential as a COPZ1‐targeted agent. BRD‐K66453893 is a screening probe originally deposited in the CTRP database with no previously reported molecular targets or biological functions. The present study represents the first identification of its potential interaction with COPZ1, based on structural modeling and molecular dynamics validation. Given the growing recognition of ferroptosis regulators as druggable nodes in chronic inflammatory diseases [36], this study proposes COPZ1 as a novel, cross‐disease therapeutic target and identifies BRD‐K66453893 as a lead compound, providing both a potential precision intervention strategy for CD, UC, and periodontitis and a valuable insight for targeting shared pathogenic mechanisms in chronic inflammatory comorbidities. In the future, COPZ1 has promising potential for clinical application as a novel biomarker. As a vesicular transport‐associated protein, COPZ1 can be conveniently detected in peripheral blood and oral saliva. Compared with invasive tissue biopsies, it offers the significant advantages of noninvasiveness, convenience, and dynamic monitorability, which are highly aligned with the clinical demands of precision stomatology and gastroenterology. Nevertheless, its clinical sensitivity and specificity still require systematic validation through large‐sample, prospective clinical cohort studies to definitively establish its application value and translational potential in real clinical practice.

In addition, the study has several limitations that should be acknowledged. First, the transcriptomic screening of DE‐FRGs was performed based on nominal *p* < 0.05 and |log2FC| > 1 without Benjamini–Hochberg correction. Therefore, the identified DE‐FRGs should be considered exploratory candidates requiring further validation. The relatively moderate statistical signals were mainly attributed to modest biological effect sizes and inherent heterogeneity across different microarray datasets. Second, our machine learning pipeline existed feature‐selection leakage: Candidate genes were screened using differential expression analysis of the entire cohort before training–test partitioning, which may lead to optimistically biased AUC results. All machine learning models were constructed using default caret package parameters without systematic hyperparameter tuning, and the lack of external independent validation further limits the generalizability and reliability of the predictive models. Third, the drug prediction analysis relied on the OncoPredict tool and CTRP database, which were originally established based on cancer cell lines. The inherent differences between tumor cell lines and inflammatory tissues may cause potential prediction bias. Accordingly, the drug screening results in this study are only preliminary in silico references and need further experimental verification. Fourth, the alterations of ferroptosis signaling in this study were merely inferred from the mRNA expression changes of Gpx4, Slc7a11, and Fth1. Corresponding protein levels, lipid peroxidation, intracellular iron accumulation, and cell viability rescue assays with ferrostatin‐1 treatment were not performed. These transcriptional alterations suggest potential changes in ferroptotic status but cannot fully confirm the occurrence of functional ferroptosis. Notably, our finding that COPZ1 knockdown increased anti‐ferroptotic transcripts is inconsistent with previous reports in glioblastoma and lung adenocarcinoma. Such divergent results may be due to cell type–specific differences and remains to be further clarified. Fifth, for scRNA‐seq analysis, although cells with zero gene counts were excluded, the median‐based grouping of COPZ1‐high and COPZ1‐low cells may still be confounded by sequencing depth and total RNA content. Several enriched functional pathways, including ribosome biogenesis, oxidative phosphorylation, and endoplasmic reticulum protein processing, are highly susceptible to library‐size variation. Moreover, CellChat‐based cell communication analysis is sensitive to cell number differences between groups, which may affect the interpretation of interaction numbers and strengths. Sixth, several public datasets were not completely independent. GSE24287 and GSE179285 were used for both CD and UC analyses, making the overlapping DE‐FRGs between IBD subtypes nonindependent. In addition, GSE10334 and GSE16134 share identical gingival tissue sources, which may contain overlapping samples. Such nonindependence may overestimate the consistency of DE‐FRGs across different inflammatory diseases. Finally, this study only conducted preliminary cellular validation, and more comprehensive in vitro functional experiments and in vivo animal studies are lacking. The detailed molecular mechanisms and biological functions of COPZ1 regulating inflammation and ferroptosis remain to be systematically explored and empirically substantiated.

## 5. Conclusion

In this study, we identified 55 common FR‐DEGs consistently dysregulated across CD, UC, and periodontitis. Integrative analysis using four complementary machine learning approaches convergently prioritized COPZ1 as the top‐ranked, cross‐disease hub gene. Experimental validation in animal models confirmed upregulation of COPZ1 protein expression in inflamed colonic tissues (DSS‐induced colitis) and periodontal tissues (ligature‐induced periodontitis). In vitro experiments demonstrated that COPZ1 is involved in the regulation of ferroptosis in macrophages. Immune infiltration and scRNA‐seq analyses demonstrated that COPZ1 expression correlates with differences in estimated infiltration patterns of macrophages, neutrophils, T cell, and B cell subsets, and that COPZ1 shows heterogeneous expression across these immune cell types, alongside its association with altered cell–cell communication in the inflammatory microenvironment. Finally, structure‐based drug prediction followed by molecular dynamics simulations identified BRD‐K66453893 as the most conformationally robust small‐molecule binder to COPZ1. Collectively, these multiomics and computational findings suggest COPZ1 as a cross‐tissue, cross‐disease regulator at the intersection of ferroptosis execution and immune microenvironment remodeling in CD, UC, and periodontitis.

## Author Contributions

**Xin Yu:** investigation, data curation, methodology, software, writing – original draft. **Yun Ruan:** investigation, methodology, writing – original draft. **Zongying Zhang:** investigation, software, validation, writing – original draft. **Jiyuan Shi:** data curation, methodology, software, validation. Xinyu Gu: investigation, visualization. **Yuyi Chen:** data curation, methodology, validation. **Mengmeng Sang:** funding acquisition, project administration, resources. **Xiaorong Zhou:** project administration. **Yan Zhou:** conceptualization, resources, supervision, writing – review & editing. **Liming Mao:** conceptualization, funding acquisition, supervision, writing – review & editing. **Xin Yu**, **Yun Ruan**, and **Zongying Zhang** contributed equally to this work.

## Funding

This study was supported by the National Natural Science Foundation of China (10.13039/501100001809) (32270919, 32470927), Jiangsu Innovative and Entrepreneurial Research Team Program (JSSCTD202348), Postgraduate Research & Practice Innovation Program of Jiangsu Province (KYCX23_3420), Nantong Social and People′s Livelihood Science and Technology Project (MS2025012), and Nantong University Special Research Fund (2025JQ013).

## Disclosure

All the authors have read and approved the final version of the manuscript.

## Ethics Statement

All the experimental procedures were approved by the Ethics Committee of the Nantong University (S20211204‐001, approved on December 4, 2021).

## Conflicts of Interest

The authors declare no conflicts of interest.

## Supporting Information

Additional supporting information can be found online in the Supporting Information section.

## Supporting information


**Supporting Information 1** Figure S1: PCA plots show the fixation before batch processing in the (a) CD, (c) UC, and (e) periodontitis datasets, and the fixation after batch processing in the (b) CD, (d) UC, and (f) periodontitis datasets. Figure S2: ROC curves of (a) CD colon samples, (b) UC colon samples, and (c) periodontitis gingival samples indicate the accuracy of gene prioritization via machine learning. Figure S3: ScRNA‐seq analysis of GSE134809. (a) Cell type annotation. (b) Bar plot showing the distribution of COPZ1‐high and COPZ1‐low cells across clusters. (c) Heatmap illustrating the distribution of 55 Co‐DEGs across different cell types. Figure S4: Drug sensitivity thermogram of (a) CD colon samples, (b) UC colon samples, and (c) periodontitis gingival samples.

**Supporting Information 2** Table S1: Ferroptosis‐related genes retrieved from FerrDB.

**Supporting Information 3** Table S2: Evaluation of batch effect removal by ComBat in CD, UC, and periodontitis datasets.

**Supporting Information 4** Table S3: Sequences of siRNAs used in this study.

**Supporting Information 5** Table S4: Primers for quantitative real‐time polymerase chain reaction.

## Data Availability

The data that support the findings of this study are available from the corresponding authors upon reasonable request.
