# Supplementary material for: Identification of COPZ1 as a Shared Candidate Ferroptosis‐Related Hub Gene in Periodontitis and Inflammatory Bowel Disease
Source: Hum Mutat. 2026 Sep 12;2026:6316527. doi: 10.1155/humu/6316527 (PMC13570568; doi:10.1155/humu/6316527)
Supplement: Supplementary file 1 [file HUMU-2026-6316527-s001.docx]

**Supplementary Figure S1.** PCA plots show the fixation before batch processing in the CD **(a)**, UC **(c)**, and periodontitis **(e)** datasets, and the fixation after batch processing in the CD **(b)**, UC **(d)**, and periodontitis **(f)** datasets.

**Supplementary Figure S2.** ROC curves of CD colon samples**(a)**, UC colon samples**(b)** and periodontitis gingival samples**(c)**, indicate the accuracy of gene prioritization via machine learning.

**Supplementary Figure S3.** ScRNA-seq analysis of GSE134809. (a) Cell type annotation. (b) Bar plot showing the distribution of COPZ1-high and COPZ1-low cells across clusters. (c) Heatmap illustrating the distribution of 55 Co-DEGs across different cell types.

**Supplementary Figure S4.** Drug sensitivity thermogram of CD colon samples **(a)**, UC colon samples **(b)** and periodontitis gingival samples **(c)**.
